# Supplementary material for: Structural effects of high laser power densities on an early bacteriorhodopsin photocycle intermediate
Source: Nat Commun. 2024 Nov 27;15:10278. doi: 10.1038/s41467-024-54422-8 (PMC11603225; doi:10.1038/s41467-024-54422-8)
Supplement: Supplementary file 1 — Supplementary Information [file 41467_2024_54422_MOESM1_ESM.pdf]

Structural effects of high laser power densities on an early bacteriorhodopsin photocycle intermediate

Quentin Bertrand, Przemyslaw Nogly, Eriko Nango, Demet Kekilli, Georgii Khusainov, Antonia Furrer, Daniel James, Florian Dworkowski, Petr Skopintsev, Sandra Mous, Isabelle Martiel, Per Börjesson, Giorgia Ortolani, Chia-Ying Huang, Michal Kepa, Dmitry Ozerov, Steffen Brünle, Valerie Panneels, Tomoyuki Tanaka, Rie Tanaka, Kensuke Tono, Shigeki Owada, Philip J. M. Johnson, Karol Nass, Gregor Knopp, Claudio Cirelli, Christopher Milne, Gebhard Schertler, So Iwata, Richard Neutze, Tobias Weinert, Jörg Standfuss

Supplementary Information

Supplementary information inventory:

Supplementary Figures 1 to 7: pages 2 to 8
Supplementary Tables 1 to 4: pages 9 to 12
Supplementary references: page 13

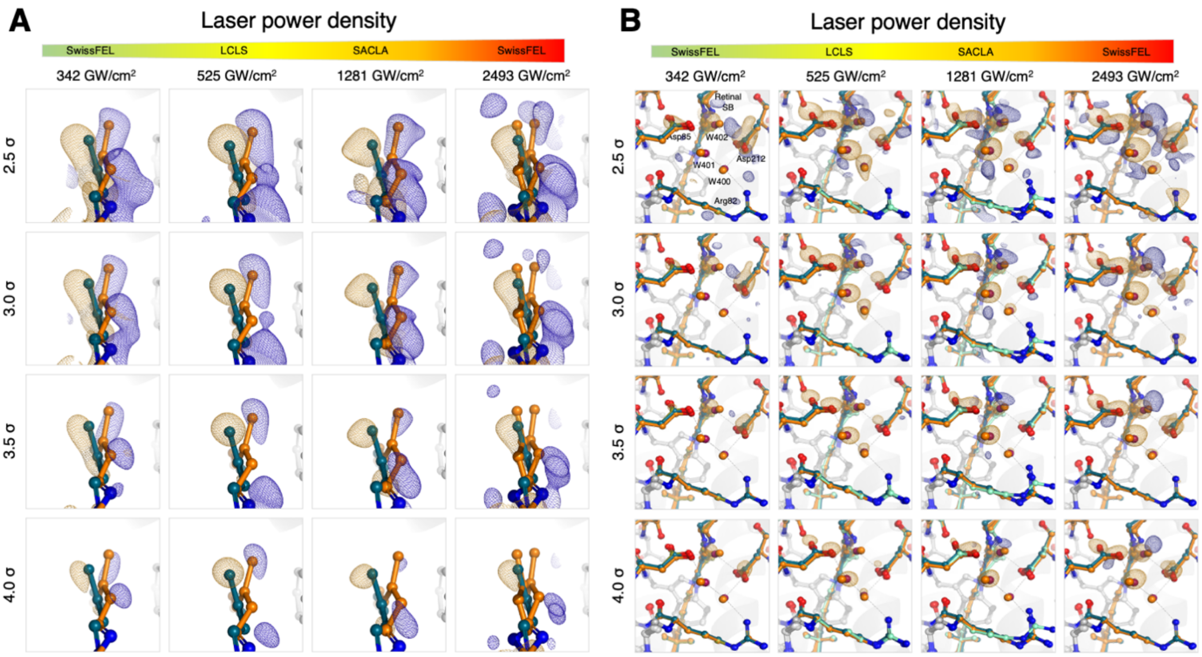

Supplementary Figure 1: Comparison at different contour levels.
View of (A) the retinal and (B) the counter-ion cluster from data collected at different laser power densities and X-ray laser facilities. As in main Figures 3 and 4, the models represented are: the refined dark model (dark green); the refined light model (light blue) and the two structures obtained at the highest laser power density (orange) for comparison. Difference electron density maps (F_{obs}(light)-F_{obs}(dark), negative density in yellow, positive density in blue) are contoured in steps of 0.5 σ. All data were truncated at 1.8 Å to allow for a better comparison between different measurements at different resolutions and facilities (i.e. lowest and highest laser power density from the same experiment at SwissFEL, with the two middle panels calculated with data from LCLS and SACLA, compare Supplementary Table 1 for crystallographic data statistics). To some extent, negative difference densities on water atoms are dependent on crystal resolution, which can be partially corrected for by truncating the data. The LCLS data have the highest resolution, followed by SACLA and SwissFEL, therefore the increase in heat signal is most obvious when comparing the outer panels (lowest and highest laser power density collected during the same experiment at SwissFEL). It is generally more difficult to compare datasets collected at different facilities with different batches of crystals, i.e. the stronger signal on Asp212 in the maps calculated from SwissFEL data. However, isomorphous differences maps peaks can arise from minuscule structural changes, particularly when the shift is less than an atoms diameter[1], and refinement against extrapolated data resolve these issues leading to very similar structures (compare Supplementary Table 3). Importantly, the observed trend of increased heterogeneity proceeds with increasing power density even when comparing data collected at three different facilities. At the lowest laser power density there is no thermal signature on other water molecules, while water 402 still moves as expected for the K-intermediate. This aligns well with the relatively modest B-factor increases in the active site observed at 1281 GW/cm^2, suggesting that the thermal energy deposited at the lowest laser power is negligible, explaining why the observed difference density map is the cleanest.

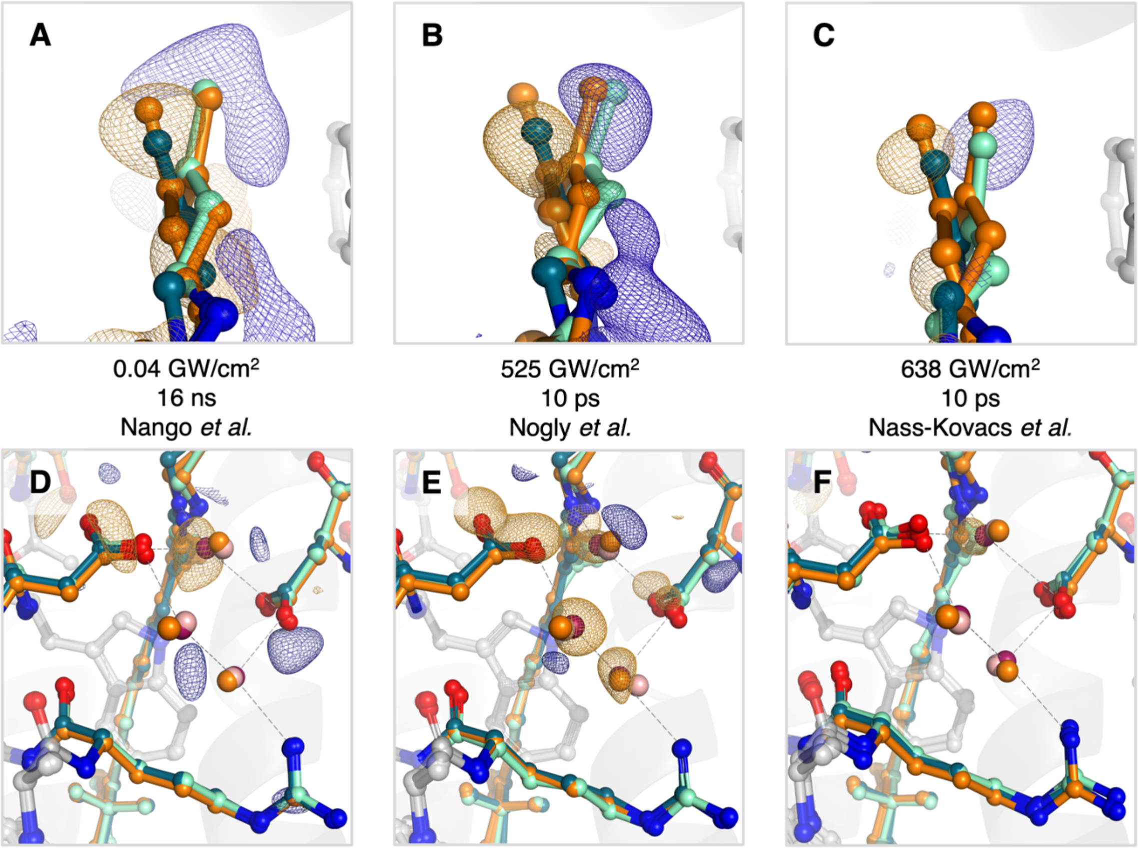

Supplementary Figure 2: **Comparison of previously published K-intermediates.** (A-C) Side by side comparison of retinal and (D-F) the counterion cluster refined using previously published data[2-4]. F_{obs}(light)-F_{obs}(dark) difference electron density maps are contoured at 3 σ. The models represented are: the refined dark model (dark green); the refined light model (light blue) and the two structures obtained at a laser power density of 2493 GW/cm^2 (orange) for comparison. While the overall structural changes are comparable between femtosecond (A+D, C+F) and nanosecond laser data (B+E), lower data quality and resolution diminishes the observation of heating effects on the counterion cluster as well as subtle shifts of the retinal. The use of custom restraints in conjunction with real space refinement using few diffraction images (C, F) resulted in a stronger retinal distortion not observed in other experiments.

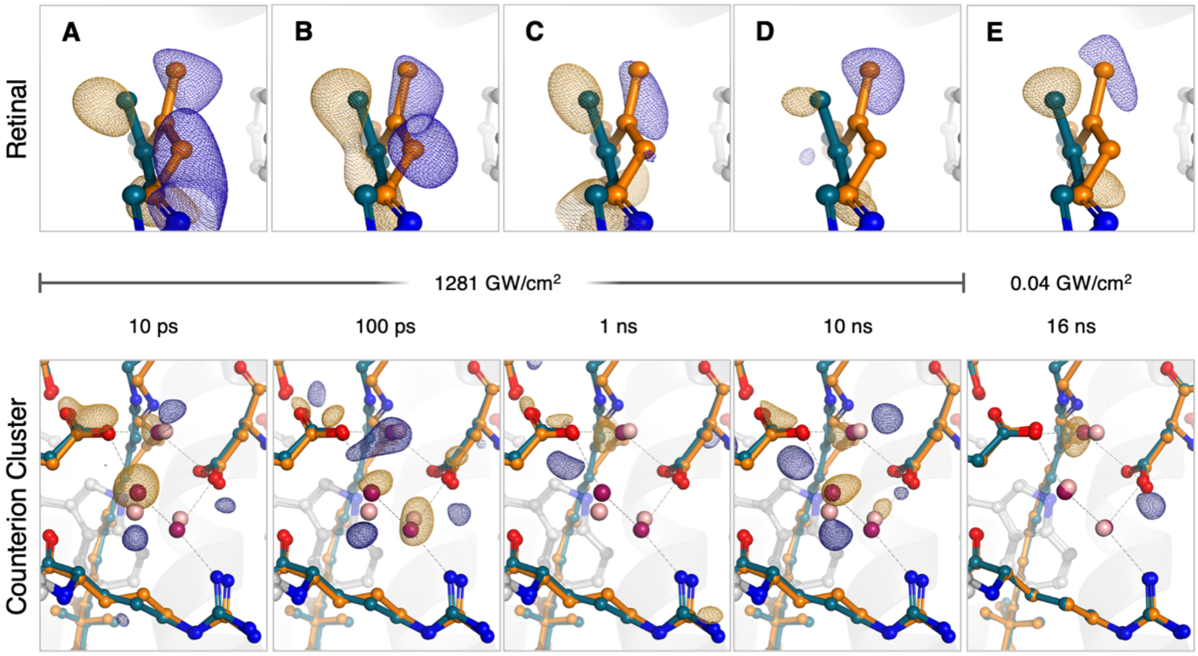

Supplementary Figure 3: Evolution of difference electron density over time.
The upper panels show a view of retinal; the lower panels show the counterion cluster. (A-D) Panels from 10 ps to 10 ns show electron difference maps (Fobs(light)-Fobs(dark), contoured at 3 σ) from datasets with the same number of images collected with a power density of 1281 GW/cm^2. Panel E shows the corresponding map obtained from re-analyzed data collected[2] with a nanosecond laser at 0.04 GW/cm^2 contoured at 3.5 σ for better comparison. Overall quality of dark as well as light data is lower for the 1 ns and 10 ns and 16 ns time delays (Supplementary Table 4). Therefore, all displayed maps were truncated to 2.3 Å resolution and no structures were refined. Negative and Positive difference maps peaks on methyl-C20 remain at comparable level throughout the time series indicative for constant levels of activation and no change in the photoproduct. Peak positions compare well to 16 ns data, though in the nanoseconds, the methyl-C20 peak begins to broaden and a shift of the Schiff base and Lys216 observed in the picoseconds starts to settle back, possibly due to the onset of L-intermediate formation. While the counterion cluster remains in motion, a general trend shows the decay of negative difference signals and confirms that the shift of Wat402 initiated in early femtoseconds[3] persists into the nanoseconds and is also present in data collected with the low laser power density of a nanosecond laser.

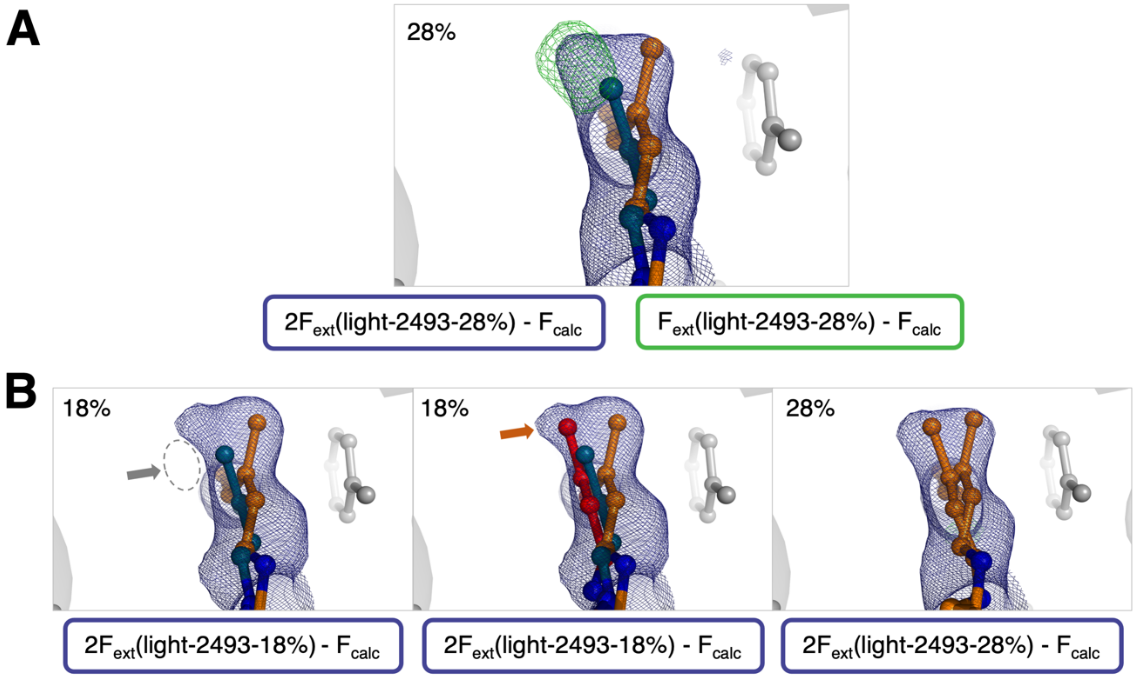

Supplementary Figure 4: Effect of activation level on retinal at the highest laser power density. (A) Extrapolated map ($2F_{ext}$(light)-F_{cal}) for the high-power density data using the determined activation level of 28%. The density cannot be fully explained by the refined retinal conformation (orange model). Residual positive density (green) suggests the presence of an additional retinal conformation situated above the dark retinal conformation (blue model). (B) Even when over-subtracting the dark model structure factors with an 18% activation level, a bulge in the density around the dark model becomes visible (grey arrow). The additional density can be explained by a retinal conformation similar to the one observed 720 ns after photoactivation by Nango *et al.*[2] (orange arrow, model shown in red). The model for the high laser power density data shown in the right panel contains two retinal conformers that together explain the density observed in the respective extrapolated map. All $2F_{ext}$(light)-F_{cal} maps are shown at 1 σ, while the F_{ext}(light)-F_{cal} map is displayed at 3 σ. The observed additional retinal conformation aligns with the L-intermediate conformation observed after 720 ns, which given the pronounced heating effects at higher laser powers appears possible, the convoluted extrapolated electron density does not allow to discern individual atomic coordinates for both isomerized retinal molecules and the model is just one potential explanation.

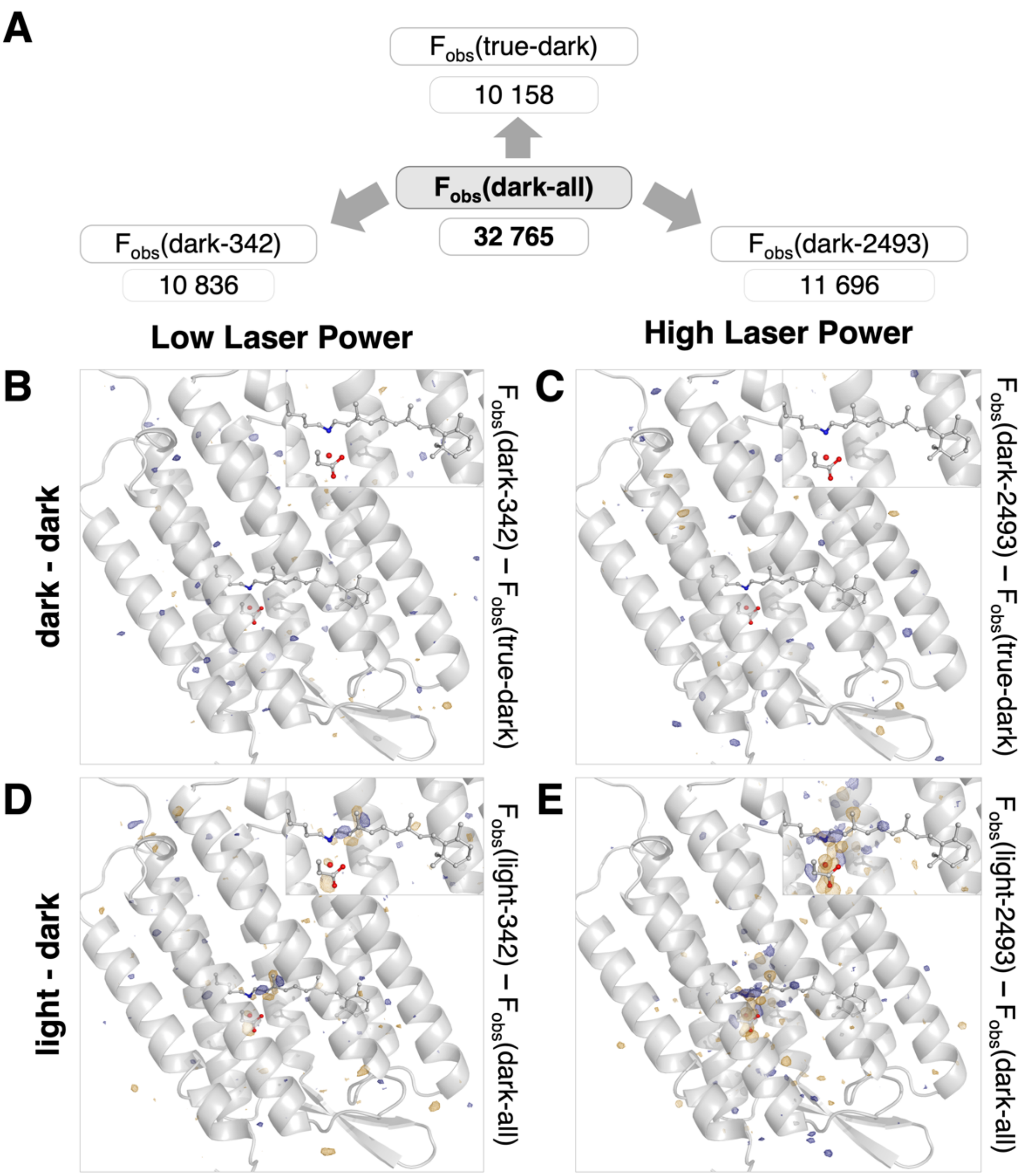

Supplementary Figure 5: Control for light contamination at low and high laser powers. To exclude that the additional retinal in the high laser power density dataset originates from insufficient spacing between interleaving dark and light shots in the time-resolved pump-probe experiment, we calculated a series of control electron difference density maps. (A) Different dark datasets available from the experiment at SwissFEL: data collected without laser (true-dark) and collected during the time-resolved experiment where dark and light images were interleaved using high (dark-2493) and low (dark-342) laser power densities, respectively. (B and C) Difference electron density maps calculated using the dark and true-dark datasets are flat, indicating the absence of light-contamination. (D and E) Difference electron density maps calculated using the respective light-activated frames from the high and low laser power density measurements are shown as comparison. Positive and negative densities are shown as blue and yellow grids contoured at 3 σ. The retinal, lysine 216, aspartic acid 212 and water 402 are represented to highlight the location of difference density.

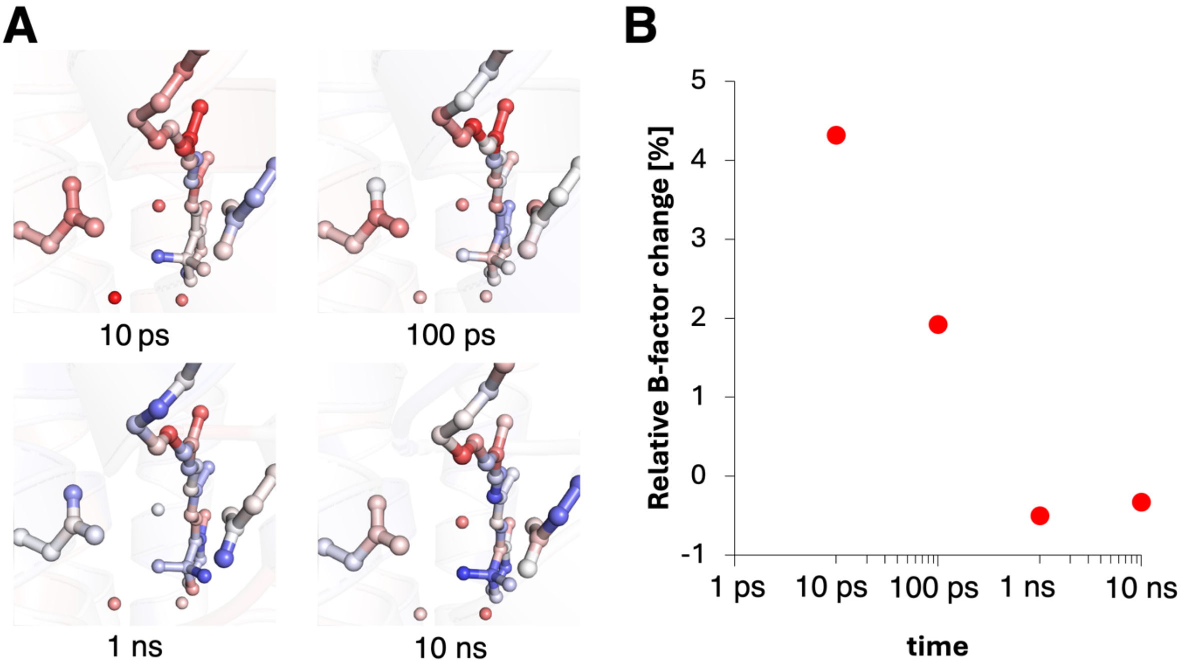

Supplementary Figure 6: Heat dissipation on the counterion cluster.
(A) Relative changes in the refined atomic B-factor on the counterion cluster and the associated retinal chromophore between laser triggered and dark data (blue=decrease, red=increase). (B) Average relative increase of the B-factor on the atoms of the counterion cluster.

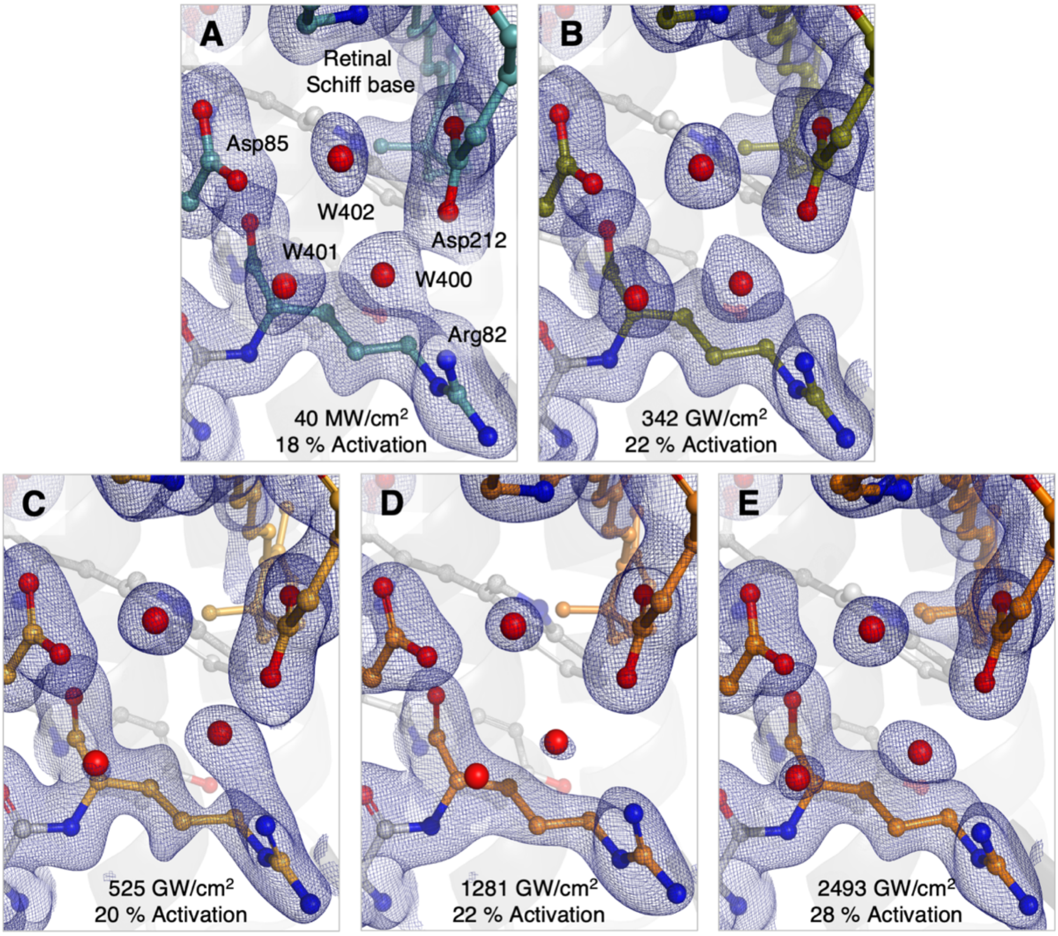

Supplementary Figure 7: Extrapolated density maps around the counterion cluster.
(A) Extrapolated map ($2F_{ext}$(light)-F_{cal}, 1 σ) calculated using reprocessed data first published by Nango *et al.*[2]. (B) Extrapolated map for data from SwissFEL collected at 342 GW/cm^2, the lowest laser power density available during commissioning. (C) Extrapolated map for data first published by Nogly *et al.*[3]. (D) Extrapolated map for data collected at 1281 GW/cm^2. (E) Extrapolated map for data at 2493 GW/cm^2, the highest laser power available at the time.

The extrapolated maps illustrate the combined effect of laser power density, spatial resolution, and activation level on the observed density in the water cluster near the retinal. Higher laser power densities (C-E) enhance the negative difference density on the water molecules seen in Supplementary Figure 1, especially on Water 401, as heat is transferred during retinal isomerization and ground-state relaxation processes. At the same time, higher resolution allows for better visualization of subtle shifts in the water molecule positions, contributing to a more pronounced difference density.

In panel (C), despite the lower laser power density, the higher spatial resolution and lower activation level (determined by retinal motion) lead to increased sensitivity to positional shifts in the water molecules. The higher resolution better captures the small vibrational displacements, resulting in lower observed extrapolated density – an “overextrapolation”.

In panel (E), the highest laser power density, combined with a higher activation level that includes an additional retinal conformation, reduces the "overextrapolation" effect.

This figure demonstrates the complex interplay between heat transfer, spatial resolution, and activation determination, showing how each factor influences the observed extrapolated density in the water cluster.

Dataset	*SACLA(Nango et al.)*		SwissFEL			*LCLS(Nogly et al.)*		SACLA	
	Dark	Light - 16 ns	Dark	Low Laser Power	High Laser Power	Dark	Light - 10 ps	Dark	Light – 10 ps
**Data Collection**									
Space group	$P6_3$								
Unit cell *a, b, c* (Å) / α , β , γ (°)	62.32, 62.32, 111.1 / 90, 90 ,120								
Indexed patterns	325 179	25 487	32 765	43 892	45 778	241 475	30 245	178 536	32 400
Activation level (%)	0	18	0	22	28	0	20	0	24
**Overall Statistics (High-Resolution Statistics)**									
Resolution (Å)	38.90 – 1.83 (1.89 – 1.83)	38.92 – 1.95 (2.00 – 1.95)	16.02 – 1.65 (1.70 – 1.65)	16.02 – 1.71 (1.77 - 1.71)	16.02 – 1.66 (1.71 – 1.66)	19.35 – 1.44 (1.48 – 1.44)	19.35 – 1.55 (1.60 – 1.55)	31.16 – 1.59 (1.64 – 1.59)	31.16 – 1.65 (1.70 – 1.65)
Total Reflections	88 655 234	6 700 561	5 605 843	6 085 565	6 970 031	61 809 503	6 905 779	63 857 212	13 705 516
Unique Reflections	21 924 (2 177)	18 140 (1 820)	30 124 (2 996)	27 072 (2 685)	29 593 (2 938)	45 078 (4 511)	35 4635 (3 534)	32 843 (3 272)	29 412 (2 913)
$<I/s(I)>$	37.43 (1.38)	12.5 (1.11)	9.20 (1.07)	9.21 (1.06)	9.94 (1.10)	22.13 (0.87)	9.19 (1.07)	15.9 (1.19)	8.2 (1.14)
CC^*	0.999 (0.878)	0.996 (0.825)	0.996 (0.833)	0.997 (0.809)	0.996 (0.838)	0.999 (0.742)	0.992 (0.729)	0.999 (0.614)	0.998 (0.633)
R_{split} (%)	2.04 (64.81)	6.32 (78.03)	9.66 (71.39)	9.5 (69.45)	8.73 (68.54)	3.01 (113.81)	8.30 (99.52)	4.23 (85.81)	8.96 (90.72)
Completeness (%)	100 (100)	100 (100)	100 (100)	100 (100)	100 (100)	100 (100)	100 (100)	100 (100)	100 (100)
Multiplicity	4043 (193)	369 (64)	186 (123)	224 (146)	235 (155)	1371 (43.1)	194 (29.6)	1944 (201)	425 (143)
**Refinement**									
Resolution Range	21.92 – 2.00	15.74 – 2.00	16.02 – 1.70	15.74 – 1.8	15.74 – 1.8	20.06 – 1.44	19.35 – 1.66	31.23 – 1.60	16.02 – 1.76
No. Reflections	16 753	16 509	27 497	22 755	22 380	44 100	27 736	32 349	22 437
R_{work} / R_{free} (%)	14.35 / 18.47	21.20 / 25.90	13.08 / 17.18	25.46 / 30.37	21.58 / 25.88	14.86 / 17.21	23.85 / 26.18	15.01 / 17.85	23.93 / 28.20
**No. Atoms**									
Protein	1855	1839	1863	1820	1848	1876	1848	1846	1854
Ligand	245	245	254	213	216	254	235	262	253
Solvent	40	35	51	68	47	46	39	45	33
**B Factors**									
Protein	43.26	31.71	35.96	23.55	35.12	32.08	26.77	34.70	53.73
Ligand	61.25	69.12	75.39	53.14	71.32	63.72	62.61	70.15	85.86
Solvent	82.45	43.88	57.06	38.46	53.00	45.56	42.80	49.48	62.00
**R.m.s Deviations**									
Bond Lengths (Å)	0.011	0.002	0.019	0.002	0.006	0.022	0.008	0.011	0.003
Bond Angles (°)	1.075	0.792	1.524	0.883	1.154	1.947	1095	1.182	0.886
**Ramachandran**									
Favoured (%)	99.56	99.56	99.56	98.22	97.79	99.12	99.12	99.56	99.12
Allowed (%)	0.44	0	0.44	1.33	1.77	0.88	0.88	0.44	0.44
Outliers (%)	0	0.44	0	0.44	0.44	0	0	0	0.44

Supplementary Table 1: Data collection and refinement statistics.

	Pulse energy (μJ)	Laser spot size (μm in 1/e²)	Pulse length (fs in 1/e²)	Peak Fluence (mJ/cm²)	Power density (GW/cm²)	Photons delivered (Ph/Mo/100fs)	High Resolution shell (Å)	Number of images
**Nogly *et al.* 10 ps**	17	95	178	93	525	10	1.6	30245
**SACLA 10 ps-10 ns**	12	141	120	154	1281	31	1.86	32400
**SwissFEL 6 ps**	1.43	80	166	57	342	9	1.7	43892
**SwissFEL 6 ps**	10.4			414	2493	61		45778
**Nango *et al.* 16 ns**	4	80	4000000	160	0.04	$8*10^{-4}$	2	25497
**Nass Kovacs *et al.* 10 ps**	5.9	99	145	93	638	15	1.8	8997

Supplementary Table 2: Summary of laser parameters for the different experiments. Values are calculated at interaction zone directly from experimental parameters. Power densities for Nogly *et al.*[3] and Nass-Kovacs *et al.*[4] have been calculated taking laser shifts of 50 µm and 25 µm, respectively, into account (Materials and Methods). The average number of photons per molecule and 100 fs is given to estimate the possibility of direct multiphoton absorption by the excited state. Real values will be significantly lower because we did not take uncertain factors into account (compare Figure 1A).

A RMSD Cα overall structure

**Dark**	/								
**0.04 GW/cm^2**	0.102	/							
**Dark**	0.12	0.141	/						
**342 GW/cm^2**	0.178	0.182	0.162	/					
**2493 GW/cm^2**	0.145	0.152	0.136	0.154	/				
**Dark**	0.11	0.15	0.134	0.226	0.181	/			
**525 GW/cm^2**	0.174	0.172	0.154	0.181	0.172	0.204	/		
**Dark**	0.099	0.138	0.12	0.223	0.175	0.075	0.201	/	
**1281 GW/cm^2**	0.127	0.138	0.134	0.169	0.151	0.166	0.171	0.148	/
	**Dark**	**0.04 GW/cm^2**	**Dark**	**342 GW/cm^2**	**2493 GW/cm^2**	**Dark**	**525 GW/cm^2**	**Dark**	**1281 GW/cm^2**

B RMSD Retinal/Lys216

**Dark**	/								
**0.04 GW/cm^2**	0.738	/							
**Dark**	0.44	0.846	/						
**342 GW/cm^2**	0.661	0.312	0.752	/					
**2493 GW/cm^2**	0.916	0.775	0.914	0.728	/				
**Dark**	0.494	0.874	0.18	0.768	1.007	/			
**525 GW/cm^2**	0.898	0.644	0.848	0.613	0.965	0.799	/		
**Dark**	0.426	0.84	0.147	0.739	1.011	0.247	0.854	/	
**1281 GW/cm^2**	0.876	0.509	0.868	0.499	0.965	0.877	0.43	0.853	/
	**Dark**	**0.04 GW/cm^2**	**Dark**	**342 GW/cm^2**	**2493 GW/cm^2**	**Dark**	**525 GW/cm^2**	**Dark**	**1281 GW/cm^2**

C RMSD Retinal/Lys216, dark vs light

Laser Power Density	0.04 GW/cm^2	342 GW/cm^2	525 GW/cm^2	1281 GW/cm^2	2493 GW/cm^2
RMSD Retinal/Lys216	0.738	0.752	0.799	0.853	0.914

Supplementary Table 3: Root mean square deviations between structures.

(A) Comparison of Cα root mean square deviations between all structures. The average difference between dark structures collected at the three X-ray laser facilities is 0.109 Å, whereas the average difference between light structures is 0.161 Å, likely because of increased errors from the extrapolation procedure. (B) Pairwise comparison of root mean square deviations for retinal and Lys216 between all light and dark structures. The average rmsd between retinal/Lys216 from all dark structures is 0.45 Å providing an error estimation between the different measurements. (C) To minimize effects from the different measurements, we focused on comparing light and dark structures collected at the same facility. These internal rmsd values between retinal/Lys216 atoms before and after isomerization remain relatively constant but start to increase with high laser power densities consistent with the heterogeneity and heating effects discussed in the main text. For an easier comparison the numerical rmsd values are supported by a blue-white-red gradient from the lowest to the highest numbers.

Dataset	SACLA								
	Full Dark	10 ps		100 ps		1 ns		10 ns	
		Dark	Light	Dark	Light	Dark	Light	Dark	Light
**Data Collection**									
Space group	$P6_3$								
Unit cell *a, b, c* (Å) / α , β , γ (°)	62.40, 62.40, 110.93 / 90, 90 ,120								
Indexed patterns	129 200	32 400	32 400	32 400	32 400	32 400	32 400	32 400	32 400
**Overall Statistics (High-Resolution Statistics)**									
Resolution (Å)	31.20 – 1.65 (1.70 – 1.65)	31.20 – 1.70 (1.76 – 1.70)	31.20 – 1.70 (1.76 – 1.70)	31.20 – 1.70 (1.76 – 1.70)	31.20 – 1.70 (1.76 – 1.70)	31.20 – 1.70 (1.76 – 1.70)	31.20 – 1.70 (1.76 – 1.70)	31.20 – 1.70 (1.76 – 1.70)	31.20 – 1.70 (1.76 – 1.70)
Total Reflections	36 119 237	14 350 726	14 145 242	12 676 170	13 405 838	10 021 415	9 933 683	11 155 767	11 212 436
Unique Reflections	29 440 (2.905)	26 930 (2 684)	26 930 (2 684)	26 930 (2 684)	26 930 (2 684)	26 930 (2 684)	26 930 (2 684)	26 930 (2 684)	26 930 (2 684)
<I/s(I)>	15.07 (1.60)	8.86 (1.46)	8.80 (1.40)	8.58 (1.11)	8.75 (1.00)	7.33 (0.60)	7.59 (0.51)	8.26 (0.80)	8.69 (0.64)
CC^*	0.999 (0.869)	0.993 (0.637)	0.998 (0.857)	0.998 (0.788)	0.998 (0.759)	0.997 (0.550)	0.998 (0.427)	0.998 (0.616)	0.998 (0.527)
R_{split} (%)	4.67 (62.06)	8.61 (65.71)	8.81 (70.67)	8.84 (91.54)	8.26 (101.29)	9.70 (178.9)	9.41 (212.69)	8.71 (136.8)	8.63 (175.46)
Completeness (%)	100 (100)	100 (100)	100 (100)	100 (100)	100 (100)	100 (100)	100 (100)	100 (100)	100 (100)
Multiplicity	1222 (238)	532 (189)	525 (184)	470 (110.6)	497 (137)	372 (63.3)	368 (42.9)	414 (64.0)	416 (36.7)

Supplementary Table 4: Data collection statistics for datasets collected with a laser power density of 1281 GW/cm^2 at different time delays.
It is obvious that the resolution of 1 ns and 10 ns data is reduced for both light and dark data due to lower crystal quality. Nevertheless, an interesting observation can be made that the data quality difference between light and dark datasets increase in the nanoseconds when the deposited energy is dissipated into the crystal.

Supplementary references:

1 Rould, M. A. & Carter, C. W., Jr. Isomorphous difference methods. *Methods Enzymol* **374**, 145-163 (2003). https://doi.org:10.1016/S0076-6879(03)74007-5

2 Nango, E. *et al.* A three-dimensional movie of structural changes in bacteriorhodopsin. *Science* **354**, 1552-1557 (2016). https://doi.org:10.1126/science.aah3497

3 Nogly, P. *et al.* Retinal isomerization in bacteriorhodopsin captured by a femtosecond x-ray laser. *Science* **361** (2018). https://doi.org:10.1126/science.aat0094

4 Nass Kovacs, G. *et al.* Three-dimensional view of ultrafast dynamics in photoexcited bacteriorhodopsin. *Nat Commun* **10**, 3177 (2019). https://doi.org:10.1038/s41467-019-10758-0
