# Supplementary material for: Structural effects of high laser power densities on an early bacteriorhodopsin photocycle intermediate
Source: Nat Commun. 2024 Nov 27;15:10278. doi: 10.1038/s41467-024-54422-8 (PMC11603225; doi:10.1038/s41467-024-54422-8)
Supplement: Supplementary file 2 — Reporting Summary [file 41467_2024_54422_MOESM2_ESM.pdf]

nature portfolio

Corresponding author(s): Tobias Weinert, Jörg Standfuss

Last updated by author(s): YYYY-MM-DD

Reporting Summary

Nature Portfolio wishes to improve the reproducibility of the work that we publish. This form provides structure for consistency and transparency in reporting. For further information on Nature Portfolio policies, see our Editorial Policies and the Editorial Policy Checklist.

Statistics

For all statistical analyses, confirm that the following items are present in the figure legend, table legend, main text, or Methods section.

n/a	Confirmed	
x		The exact sample size (n) for each experimental group/condition, given as a discrete number and unit of measurement
	x	A statement on whether measurements were taken from distinct samples or whether the same sample was measured repeatedly
x		The statistical test(s) used AND whether they are one- or two-sided<br>*Only common tests should be described solely by name; describe more complex techniques in the Methods section.*
x		A description of all covariates tested
x		A description of any assumptions or corrections, such as tests of normality and adjustment for multiple comparisons
x		A full description of the statistical parameters including central tendency (e.g. means) or other basic estimates (e.g. regression coefficient) AND variation (e.g. standard deviation) or associated estimates of uncertainty (e.g. confidence intervals)
x		For null hypothesis testing, the test statistic (e.g. F, t, r) with confidence intervals, effect sizes, degrees of freedom and P value noted<br>*Give P values as exact values whenever suitable.*
x		For Bayesian analysis, information on the choice of priors and Markov chain Monte Carlo settings
x		For hierarchical and complex designs, identification of the appropriate level for tests and full reporting of outcomes
x		Estimates of effect sizes (e.g. Cohen's d, Pearson's r), indicating how they were calculated

Our web collection on statistics for biologists contains articles on many of the points above.

Software and code

Policy information about availability of computer code

Data collection	Data collection was done at FEL endstations at SwissFEL Alvra and SACLA BL3. All necessary software is made available their after a successful beamtime proposal.
Data analysis	Collected data were processed with Crystfel 0.9.1. Isomorphous difference maps and extrapolated maps were created with Xtrapol8 (1.0 first release). Models were then refined with phenix.refine 1.20_4459. All references are included in the material and methods section. The code used for the B-factor normalization is available on Figshare.

For manuscripts utilizing custom algorithms or software that are central to the research but not yet described in published literature, software must be made available to editors and reviewers. We strongly encourage code deposition in a community repository (e.g. GitHub). See the Nature Portfolio guidelines for submitting code & software for further information.

Data

Policy information about availability of data

All manuscripts must include a data availability statement. This statement should provide the following information, where applicable:

- Accession codes, unique identifiers, or web links for publicly available datasets
- A description of any restrictions on data availability
- For clinical datasets or third party data, please ensure that the statement adheres to our policy

Coordinates and structure factors for dark state bacteriorhodopsin have been deposited on the PDB database server under accession codes 9F9D [http://doi.org/10.2210/pdb9F9D/pdb] (SwissFEL) and 9F9I [http://doi.org/10.2210/pdb9F9I/pdb] (SACLA, fs laser). Models refined against extrapolated data, extrapolated

structure factors, and light data used for data extrapolation have been deposited under accession codes: 9F9E [http://doi.org/10.2210/pdb9F9E/pdb] (342 GW/cm2), 9F9J [http://doi.org/10.2210/pdb9F9J/pdb] (1281 GW/cm2), and 9F9F [http://doi.org/10.2210/pdb9F9F/pdb] (2493 GW/cm2). Reprocessed dark and light data from11 and the respective structures have been deposited under 9F9B [http://doi.org/10.2210/pdb9F9B/pdb] (dark) and 9F9C [http://doi.org/10.2210/pdb9F9C/pdb] (0.04 GW/cm2). Reprocessed dark and light data from16 and the respective structures have been deposited under 9F9G [http://doi.org/10.2210/pdb9F9G/pdb] (dark) and 9F9H [http://doi.org/10.2210/pdb9F9H/pdb] (525 GW/cm2). The code used to analyze the difference of B-factor between light and dark dataset in our SACLA, fs laser data is deposited on figshare (DOI: [https://doi.org/10.6084/m9.figshare.27332034.v2]). Source data for Figures 2, 5 and Supplementary Figure 6 are provided in a Source Data file.

Research involving human participants, their data, or biological material

Policy information about studies with human participants or human data. See also policy information about sex, gender (identity/presentation), and sexual orientation and race, ethnicity and racism.

Reporting on sex and gender	This study is not involving human participants.
Reporting on race, ethnicity, or other socially relevant groupings	This study is not involving human participants.
Population characteristics	This study is not involving human participants.
Recruitment	This study is not involving human participants.
Ethics oversight	This study is not involving human participants.

Note that full information on the approval of the study protocol must also be provided in the manuscript.

Field-specific reporting

Please select the one below that is the best fit for your research. If you are not sure, read the appropriate sections before making your selection.

[x] Life sciences   [ ] Behavioural & social sciences   [ ] Ecological, evolutionary & environmental sciences

For a reference copy of the document with all sections, see nature.com/documents/nr-reporting-summary-flat.pdf

Life sciences study design

All studies must disclose on these points even when the disclosure is negative.

Sample size	n/a
Data exclusions	n/a
Replication	n/a
Randomization	n/a
Blinding	n/a

Reporting for specific materials, systems and methods

We require information from authors about some types of materials, experimental systems and methods used in many studies. Here, indicate whether each material, system or method listed is relevant to your study. If you are not sure if a list item applies to your research, read the appropriate section before selecting a response.

Materials & experimental systems

n/a	Involved in the study	
x		Antibodies
x		Eukaryotic cell lines
x		Palaeontology and archaeology
	x	Animals and other organisms
x		Clinical data
x		Dual use research of concern
x		Plants

Methods

n/a	Involved in the study	
x		ChIP-seq
x		Flow cytometry
x		MRI-based neuroimaging

Animals and other research organisms

Policy information about studies involving animals; ARRIVE guidelines recommended for reporting animal research, and Sex and Gender in Research

Laboratory animals	The study doesn't involve Laboratory animals.
Wild animals	The study doesn't involve wild animals.
Reporting on sex	The study doesn't involve animals.
Field-collected samples	The study doesn't involve field collected samples.
Ethics oversight	n/a

Note that full information on the approval of the study protocol must also be provided in the manuscript.

Plants

Seed stocks	n/a
Novel plant genotypes	n/a
Authentication	n/a
